# A Novel Dual Signaling Axis for NSP 5a3a induced apoptosis in Head and Neck Carcinoma

**DOI:** 10.18632/oncotarget.306

**Published:** 2011-12-14

**Authors:** Luca D'Agostino, Antonio Giordano

**Affiliations:** ^1^ Sbarro Institute for Cancer Research and Molecular Medicine & Department of Biology, College of Science and Technology Temple University, 1900 North 12th street room 431, Philadelphia PA 19122

**Keywords:** NSP 5a3a, Head and Neck Carcinoma, TNFR-1 signaling pathway

## Abstract

NSP 5a3a is a novel structural protein found to be over-expressed in certain cancer cell lines in-vitro such as Hela, Saos-2, and MCF-7 while barely detectable levels in normal body tissues except for Testis. This particular isoform has been known to interact with cyto- nuclear proteins B23, known to be involved in multi-faceted cellular processes such as cell division, apoptosis, ribosome biogenesis, and rRNA processing, as well as with hnRNP-L, known to be involved with RNA metabolism and rRNA processing. A previous preliminary investigation of NSP 5a3a as a potential target in Head and Neck Carcinoma revealed a novel p73 dependent mechanism through which NSP 5a3a induced apoptosis in Head and Neck cell lines when over-expressed in-vitro. Our present investigation further elucidated a novel dual axis signaling point by which NSP 5a3a induces apoptosis in Head and Neck cell line HN30 through p73-DAXX and TRAF2-TRADD. Interestingly, this novel mechanism appears independent of canonical caspases involved in the intrinsic mitochondrial pathway as well as those in the death receptor pathway thru TRAF2 and TRADD.

## INTRODUCTION

Head and Neck squamous cell carcinomas (HNSSCs) being the fifth or sixth most common form of cancer worldwide depending on sources [8-9] has shown an increasing incidence with greater than 40,000 cases diagnosed each year in the United States and approximately 500,000 globally [10] though in the last forty years there has been no significant improvement in overall mortality and survival from this aggressive form of cancer [11-12]. Typically, the 5-year survival rates have been around 50% in the past twenty years, due to the lack of positive prognosis and reliable tumor markers for this pathological and biological diverse type of cancer hence its diverse clinical profiles and treatment [13]. Usually, general treatment involves surgery or combination of radiation and chemotherapy. Most patients will suffer from recurrences, whether locally or regionally, being from primary sites or becoming metastatic and spreading throughout the lymph nodes even still after surgery and or in combination with chemotherapy, will experience within two years and with significant decrease in overall survival by a half [14-16]. HNSSC can originate from various parts of the upper digestive/oral tract being and including larynx, oral and nasal cavities, pharynx, and esophagus [17]. HNSSC's can occur from common carcinogens found in tobacco products as well as in combination or not with of alcohol consumption [18-19] though there are cases of HNSSC where the cause is viral being from HPV (Human Papilloma virus) in about 30% of cases [20]. Thus, given the general poor prognosis and high risk of cancerous recurrences even from secondary primary tumor sites, there is push through development of novel biologic therapies including gene therapy to promote better tumor growth control or regression with higher survival rates. Such strategies have included p53 based adenoviral gene therapy [21] as well as Fas ligand adenoviral gene therapy [22].

Apoptosis also known as programmed cell death or cell suicide is a critical complex intrinsic cellular process involved in normal tissue homeostasis and development. This highly evolutionary conserved process occurs not only in normal cellular and tissue physiology but has been implicated in various pathological diseases such as cancer, autoimmune diseases, and dementia [23-29]. Typically, apoptosis is characterized by distinct morphological and biochemical features which include shrinkage of the cell followed by cleavage of cytoskeletal components such as actin and lamins, nuclear condensation and DNA fragmentation, ending with membrane blebbing and apoptotic bodies in late stage apoptosis [30-32].

Apoptosis can be initiated either through extrinsic or intrinsic signaling pathways which involve the death receptor ligand complex and mitochondria, respectively. Activation of the extrinsic pathway usually involves the tumor necrosis family (TNF) receptor superfamily including TNFR1, TRAIL-R1, TRAIL-R2, or CD95 (APO-1-Fas) involving its trimeric membrane receptor formation upon contact with its appropriate extracellular ligand and recruitment of adaptor molecules [33]. In the case of CD95 and TRAIL induced apoptosis, there is recruitment of FADD adaptor molecule and caspase 8 which becomes activated and cleaves downstream effector caspases such as caspase 3, 6, and 7 depending on the cell type or there is involvement of the mitochondria in a positive feedback loop thru cleavage of Bid [34-35]. While, in the case of TNF-R1 induced apoptosis, there is recruitment of adaptor molecules TRADD with FADD, or with RAIDD, which can lead to caspase 8 activation followed by subsequent cleavage and activation of downstream effector caspases such as caspase 3, 6, and 7 or caspase 2 activation converging on the same activation of effector caspases as mentioned before [36-38]. The intrinsic pathway also known as the mitochondrial pathway, involves the release of apoptotic factors from the mitochondrial intermembrane space such as apoptosis inducing factor (AIF), cytochrome c, or Smac/DIABLO. The release of cytochrome into the cytoplasm allows the formation of an apoptosome complex formed by cytochrome c, Apaf-1, and caspase 9 which subsequently activates caspase 3 [39-41]. Whereas SMAC/DIABLO help enhance caspase activation by inhibiting anti-apoptotic factors such as IAPs (inhibitors of apoptosis proteins) while AIF causes changes in DNA condensation [42-44]. Both pathways can be interconnected at different points such as in the case of caspase 8, where it can initiate the mitochondrial pathway by by cleavage of BID, leading to BAX/BAK dependent cytochrome c release from the mitochondria and apoptosome formation resulting in caspase 9 activation [44-45]. Then, subsequent caspase activation of caspases 3 and 7 can occur with further activation of caspases of 2 and 6. Though, it has been noted that when particular caspases are impaired functionally or not expressed, other caspases along the cascade can continue the processing to downstream effectors [46].

TRAF2 (tumor necrosis factor receptor associated factor) is a member of a highly evolutionary conserved family of adaptor proteins (TRAFs) which have been found to participate in a multitude of biological processes such as: immunity, embryonic development, stress responses and bone metabolism being mediated thru TRAFs signaling leading to cell proliferation, differentiation, and apoptosis [47]. The biological effects caused by the TRAF signaling, is possible through the activation of NF-kB and AP-1 pathways which lead usually to cell survival and apoptosis, respectively [48-50] though there is also evidence of NF-kB and AP-1 pathways being involved in apoptosis and cell proliferation as well, respectively [51-52]. Upon TNFR1 stimulation, depending on cellular microenvironment and stimuli, TRAF2 can be recruited to form a complex with another adaptor protein known as TRADD which associates with the death domain regions of the trimerized TNFR1 through its own intracellular death domains, also being able to recruit other signaling molecules such as FADD and RIP [53-54]. Depending on which signaling complex is released from TNFR1, there can be downstream activation of NF-kB through TRAF2/RIP complex resulting in pro-survival genes or even AP-1 activation by TRAF2 through upstream activation of ASK1/JNK leading to apoptosis. Should the levels of TRAF2 be deficient in the cytoplasm or absent along with limited accessibility of anti-apoptotic proteins c-IAP and FLICE, then the TRADD/FADD along with caspase 8 can initiate downstream caspase activation with subsequent apoptosis [54-57].

P73, a transcription factor belonging to the p53 family, is known to have shared and distinct functions with other p53 members, though all involved in regulating the fate of cells between cell growth and apoptosis during normal development, differentiation, and cellular stress [58-60], it has been shown to be specifically involved in biological processes such as neurogenesis, immunity and negative regulation of p53 activity [61-63]. P73 in common with other p53 members can bind and transcriptionally differently activate p53 target genes which can be involved in apoptosis and cell cycle arrest. Depending on the particular p53 member that is expressed and which isoforms in that particular tissue or cells will determine the overall fate [64-65]. P73 can exist in multiple isoforms being either amino-terminal or carboxy-terminal splice variants of the full length Tap73 isoform in which the full length and carboxy-terminal truncated isoforms have been associated with pro-apoptotic functions along with cell growth arrest and tumor suppression while the truncated amino terminal isoforms ∆Np73 have been associated with primarily anti-apoptotic functions as well as having negative regulatory activities on p53 and TAp73 isoforms [65-67]. Different modes of action have been attributed to p73 in the induction of apoptosis, which have primarily been linked to either DNA damage or cytosolic stress. Once activated and depending on the stimuli, p73 particularly TAp73 can induce apoptosis through ER stress via the transactivation of Scotin, or can activate the mitochondrial intrinsic pathway through transactivation of PUMA with subsequent translocation and activation of BAX, as well as activate the death receptor pathway [68]. Interestingly, in regards to the activation of the death receptor pathway, p73 has been shown to have transcriptional activity in the sensitization of cells to apoptosis through a Fas mediated caspase dependent mechanism [69] as well as being involved in ASK1/JNK signaling leading to apoptosis [70-71], by which this pathway has been linked to Fas signaling through DAXX which has been shown to interact with p73 [72].

NSP 5a3a along with three other splice variants isoforms: NSP 5b3a, 5a3b, and 5b3b were identified having slight homology to DNA repair Sbc proteins and SMC (structural maintenance chromosome) proteins while higher homology to Specc1 and Cytospin A renal carcinoma antigen was observed whose functions are functions are yet to characterized. These NSP isoforms having features of Spectrin like repeat proteins suggests they may be novel members of this superfamily of proteins which can be involved in a plethora of cellular activities ranging from intracellular structural integrity in the cytoskeleton and matrix in both the cytoplasm and nucleus [73-74] to intracellular trafficking [75], maintenance of organelle function, integrity [75], and endocytosis [76], nuclear reassembly in Telophase [77], to being scaffolding proteins with other cytoskeletal proteins involved in signal transduction pathways [78].

The NSP 5a3a isoform was found to be expressed differently in-vitro, while moderate in some other showed higher levels of expression such as: HT-29, MCF-7, Hela, HN30, Saos-2, CEM, and H23 [1]. NSP 5a3a was shown to interact with B23, a multi-functional nucleolar in Hela cells both in cycling and in dividing cells. This suggested a likely involvement of NSP 5a4a during cell division with B23 and as well as other B23 functions pathways [2]. Subsequently, we showed and verified that NSP 5a3a, B23, and hnRNP-L (heterogeneous ribonuclearprotein) interacted with each other in both MCF-7 (breast adenocarcinoma) and MCF-12A (normal breast) cell lines though in a both compartment and cell-type specific manner [4]. This novel interaction between NSP 5a3a with B23 and hnRNP-L raised the possibility of implicating NSP 5a3a in RNA metabolism and RNA processing both of which B23 and hnRNP-L have been confirmed to have important roles involving RNA processing and regulation [5-6].

Our previous study had demonstrated that over-expression of NSP 5a3a induced significant apoptosis in head and neck carcinoma cell lines in particular in HN30 being p53 independent yet involving a novel p73 dependent mechanism. Thus, we had proposed NSP 5a3a as a potential therapeutic target to be investigated further, since it could have potential in a site-directed manner to treat cancer types that behave through a similar p73 dependent mechanism as found in the head and neck carcinoma cell lines we investigated previously [7]. We further elucidated this novel p73 mechanism thru NSP 5a3a showing the involvement of TNF associated proteins TRAF2 and TRADD along with the TGF-beta/FAS associated apoptotic protein DAXX.

## RESULTS

### Morphological and Immunofluorescence analysis of HN30 over-expressing NSP 5a3a

**Figure 1:**
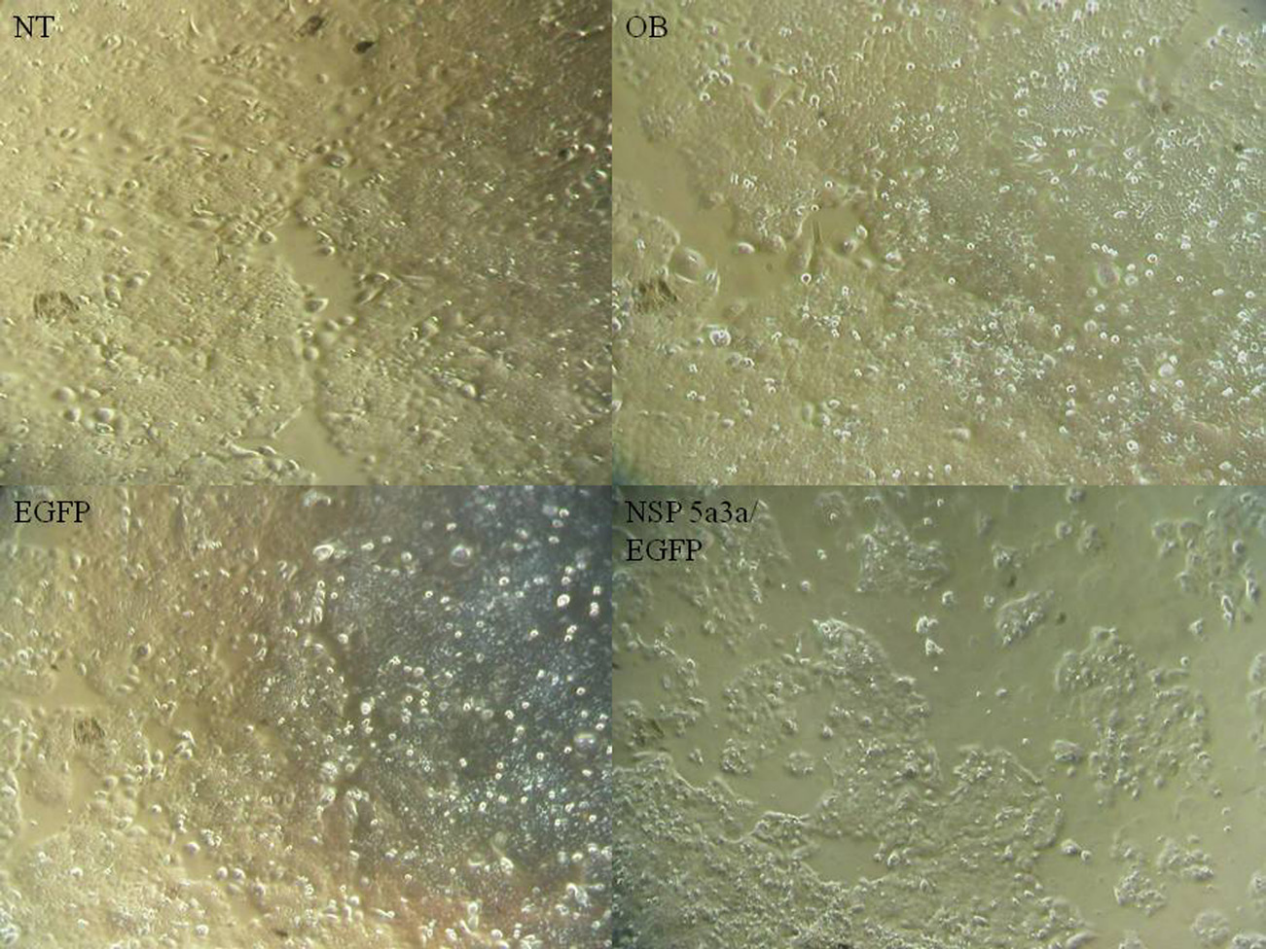
Morphological observation of HN30 cells 3 days post-transfection NT: non-treated, OB: only buffer, EGFP: only pcDNA3.1/CT-GFP vector, NSP 5a3a/EGFP: pcDNA 3.1/CT-GFP and pcDNA 3.0 NSP 5a3a.

Asynchronous HN30 cells were transfected with pcDNA3.0 NSP 5a3a vector along with controls after which images of the cells were taken three days post-transfection and prepared for immune-staining to assess the effect of NSP 5a3a over-expression on protein distribution of NSP 5a3a, p73, TRAF2, and TRADD. Images of HN30 cells three days post-transfection revealed that treated cells with NSP 5a3a seemed more stressed with appearance of floating cells and fewer attached cells to the plate surface indicative of possible apoptosis (Fig. 1). Controls were seemingly still confluent and well attached to plate surface (Fig. 1.). Immunostaining for NSP 5a3a in non-treated cells localized NSP 5a3a to mostly the nucleus while in cells over-expressing NSP 5a3a, there seemed to be a greater distribution into the cytoplasm along with a seemingly increase staining in the nucleus as well as the presence of possible protein aggregates containing NSP 5a3a with staining near and in likely association with apoptotic bodies. The cells over-expressing NSP 5a3a showed signs of apoptosis such as horse-shoe appearance of the nucleus and evidence of apoptotic bodies and blebs as indicated from the DAPI staining (Fig. 3). Immunostaining for p73 in non-treated cells localized p73 to mostly the nucleus with slight cytoplasmic staining while in cells over-expressing NSP 5a3a, there seemed to be a more diffuse distribution of p73 into the cytoplasm (Fig. 4). Immunostaining for TRAF2 in non-treated cells localized TRAF2 to both a diffuse localization in the cytoplasm and nucleus while in cells over-expressing NSP 5a3a, there seemed to be an increase of localization of TRAF2 into the cytoplasm as well as in association with protein bodies or aggregates as well as staining near and in likely association with apoptotic bodies (Fig. 5). Finally, immunostaining for TRADD in non-treated cells localized TRADD to mostly the nucleus while in cells over-expressing NSP 5a3a, there seemed to be an increase in the localization of TRADD in the cytoplasm, nucleus, and also in association with protein bodies or aggregates along with staining near and in likely association of apoptotic bodies (Fig. 6).

**Figure 2:**
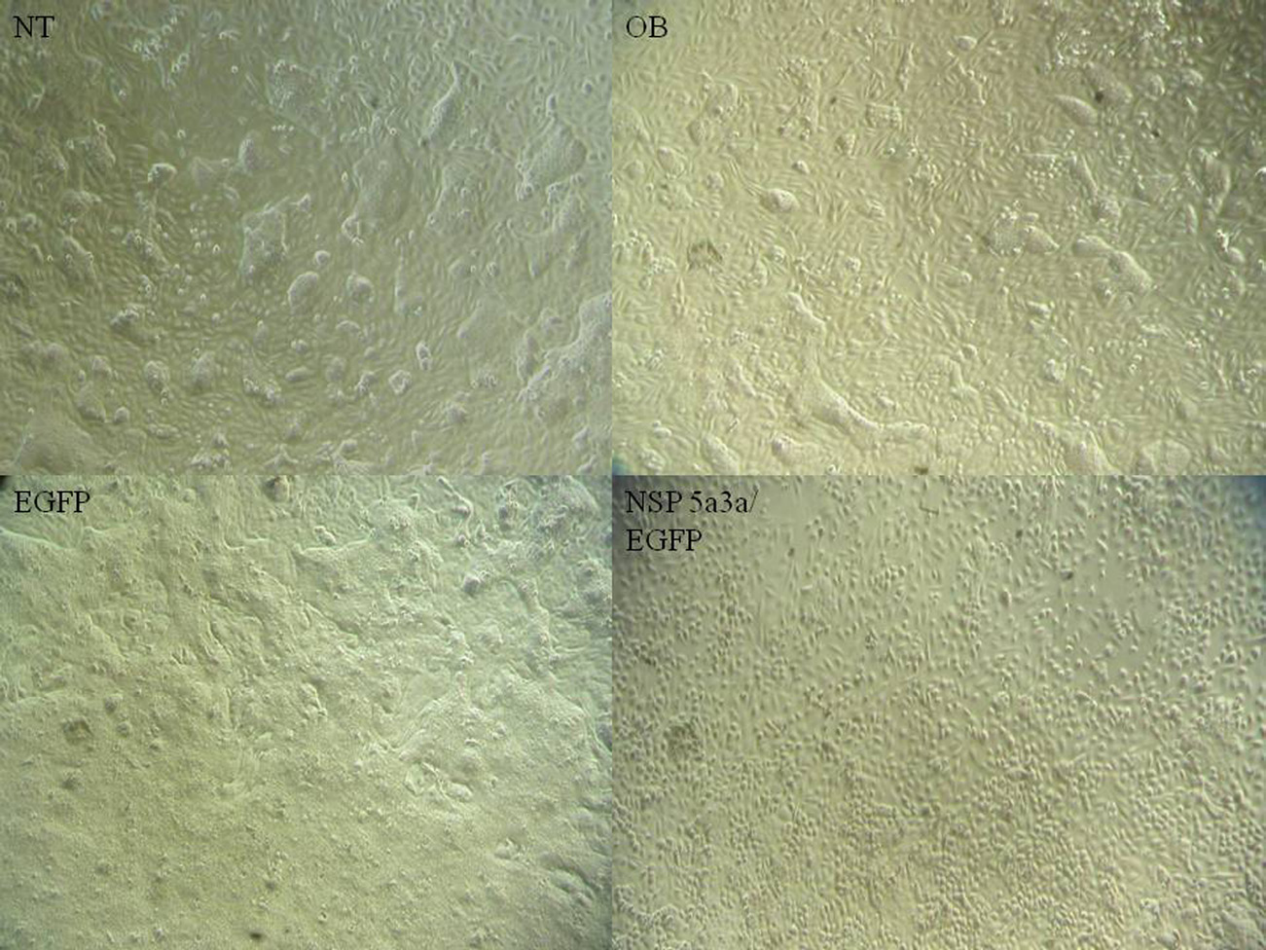
Morphological observation of MCF-12a cells 3 days post-transfection NT: non-treated, OB: only buffer, EGFP: only pcDNA3.1/CT-GFP vector, NSP 5a3a/EGFP: pcDNA 3.1/CT-GFP and pcDNA 3.0 NSP 5a3a.

**Figure 3:**
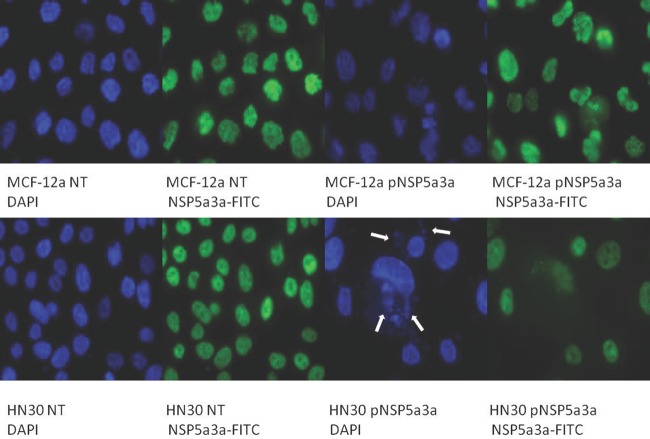
Immunostaining of HN30 and MCF-12a cells for NSP 5a3a 3 days post-transfection MCF-12a NT (DAPI): non-treated MCF-12a DAPI stained, MCF-12a NT (NSP 5a3a-FITC): non-treated MCF-12a FITC stained for NSP 5a3a, MCF-12a pNSP 5a3a (DAPI): MCF-12a transfected with pcDNA 3.0 NSP 5a3a DAPI stained, MCF-12a pNSP 5a3a (NSP 5a3a-FITC): MCF-12a transfected with pcDNA 3.0 NSP 5a3a FITC stained for NSP 5a3a, HN30 NT (DAPI): non-treated HN30 DAPI stained, HN30 NT (NSP 5a3a-FITC): non-treated HN30 FITC stained for NSP 5a3a, HN30 pNSP 5a3a (DAPI): HN30 transfected with pcDNA 3.0 NSP 5a3a DAPI stained, HN30 pNSP 5a3a (NSP 5a3a-FITC): HN30 transfected with pcDNA 3.0 NSP 5a3a FITC stained for NSP 5a3a. White arrows indicate apoptotic bodies.

**Figure 4:**
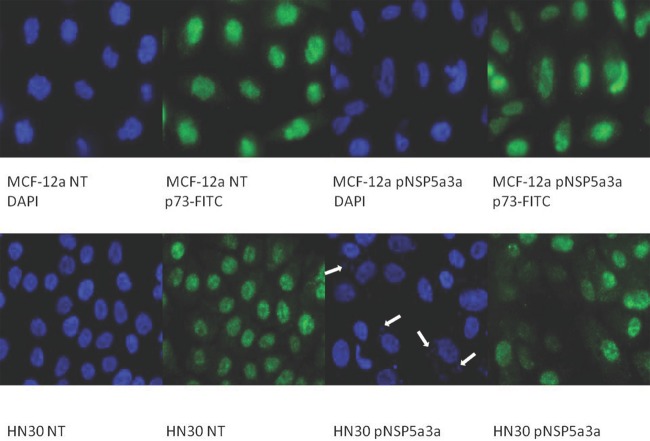
Immunostaining of HN30 and MCF-12a cells for p73 3 days post-transfection MCF-12a NT (DAPI): non-treated MCF-12a DAPI stained, MCF-12a NT (p73-FITC): non-treated MCF-12a FITC stained for p73, MCF-12a pNSP 5a3a (DAPI): MCF-12a transfected with pcDNA 3.0 NSP 5a3a DAPI stained, MCF-12a pNSP 5a3a (p73-FITC): MCF-12a transfected with pcDNA 3.0 NSP 5a3a FITC stained for p73, HN30 NT (DAPI): non-treated HN30 DAPI stained, HN30 NT (p73-FITC): non-treated HN30 FITC stained for p73, HN30 pNSP 5a3a (DAPI): HN30 transfected with pcDNA 3.0 NSP 5a3a DAPI stained, HN30 pNSP 5a3a (p73-FITC): HN30 transfected with pcDNA 3.0 NSP 5a3a FITC stained for p73. White arrows indicate apoptotic bodies.

**Figure 5:**
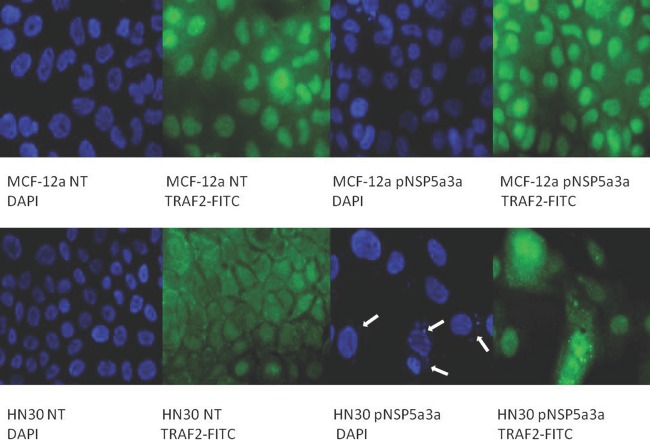
Immunostaining of HN30 and MCF-12a cells for TRAF2 3 days post-transfection MCF-12a NT (DAPI): non-treated MCF-12a DAPI stained, MCF-12a NT (TRAF2-FITC): non-treated MCF-12a FITC stained for TRAF2, MCF-12a pNSP 5a3a (DAPI): MCF-12a transfected with pcDNA 3.0 NSP 5a3a DAPI stained, MCF-12a pNSP 5a3a (TRAF2-FITC): MCF-12a transfected with pcDNA 3.0 NSP 5a3a FITC stained for Traf-2, HN30 NT (DAPI): non-treated HN30 DAPI stained, HN30 NT (TRAF2-FITC): non-treated HN30 FITC stained for TRAF2, HN30 pNSP 5a3a (DAPI): HN30 transfected with pcDNA 3.0 NSP 5a3a DAPI stained, HN30 pNSP 5a3a (TRAF2-FITC): HN30 transfected with pcDNA 3.0 NSP 5a3a FITC stained for TRAF2. White arrows indicate apoptotic bodies.

**Figure 6:**
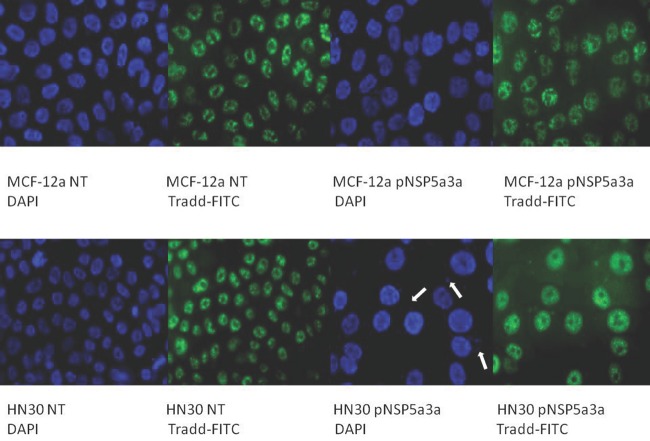
Immunostaining of HN30 and MCF-12a cells for TRADD 3 days post-transfection MCF-12a NT (DAPI): non-treated MCF-12a DAPI stained, MCF-12a NT (TRADD-FITC): non-treated MCF-12a FITC stained for TRADD, MCF-12a pNSP 5a3a (DAPI): MCF-12a transfected with pcDNA 3.0 NSP 5a3a DAPI stained, MCF-12a pNSP 5a3a (TRADD-FITC): MCF-12a transfected with pcDNA 3.0 NSP 5a3a FITC stained for Tradd, HN30 NT (DAPI): non-treated HN30 DAPI stained, HN30 NT (TRADD-FITC): non-treated HN30 FITC stained for TRADD, HN30 pNSP 5a3a (DAPI): HN30 transfected with pcDNA 3.0 NSP 5a3a DAPI stained, HN30 pNSP 5a3a (TRADD-FITC): HN30 transfected with pcDNA 3.0 NSP 5a3a FITC stained for TRADD. White arrows indicate apoptotic bodies.

**Figure 7:**
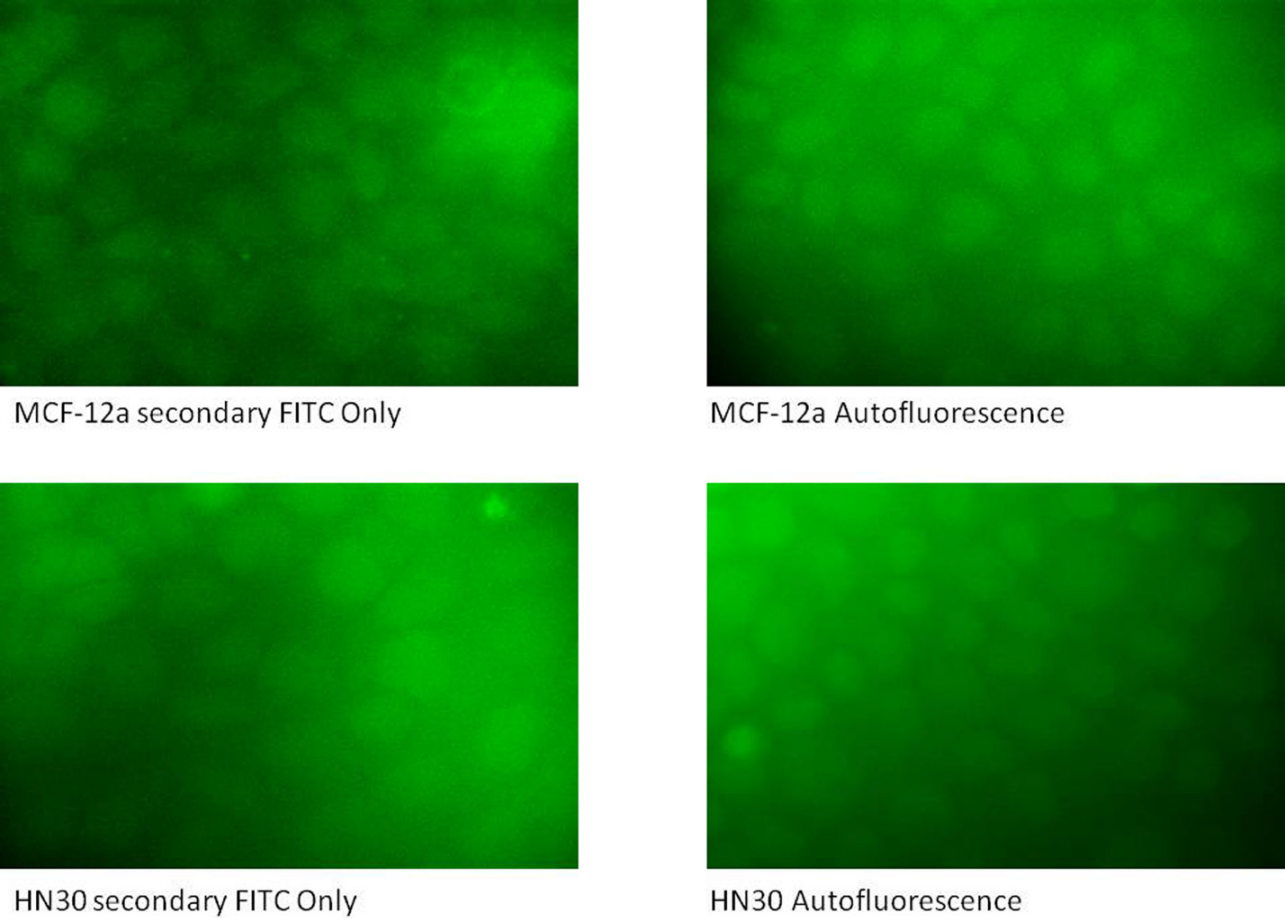
Immunostaining controls of HN30 and MCF-12a cells for secondary anti-rabbit FITC and autofluorescence

### Morphological and immunofluorescence analysis of MCF-12a over-expressing NSP 5a3a

Asynchronous MCF-12a cells were transfected with pcDNA3.0 NSP 5a3a vector along with controls after which images of the cells were taken three days post-transfection and prepared for immune-staining to assess the effect of NSP 5a3a over-expression on protein distribution of NSP 5a3a, p73, TRAF2, and TRADD. Images of MCF-12a cells three days post-transfection revealed that treated cells with NSP 5a3a seemed quite confluent and well attached to the plate surface comparable to the controls (Fig. 2). Immunostaining for NSP 5a3a in non-treated cells localized NSP 5a3a to mostly the nucleus while in cells over-expressing NSP 5a3a, there seemed to be a similar distribution to mostly nuclear and nuclear integrity of cells over-expressing NSP 5a3a showed no evident signs of cellular membrane stress as compared to the non-treated cells (Fig. 3). Immunostaining for p73 in non-treated cells localized p73 to mostly the nucleus while in cells over-expressing NSP 5a3a, there seemed to be a more diffuse localization of p73 into the cytoplasm while still being quite nuclear (Fig. 4). Immunostaining for TRAF2 in non-treated cells localized TRAF2 to both a diffuse localization in the cytoplasm and nucleus while in cells over-expressing NSP 5a3a, there seemed to be an increase of localization of TRAF2 into the nucleus while still appearing in the cytoplasm (Fig. 5). Finally, immunostaining for TRADD in non-treated cells localized TRADD to mostly the nucleus while in cells over-expressing NSP 5a3a, there seemed to be a more diffuse localization of TRADD into the cytoplasm while still being localized in the nucleus (Fig. 6).

### Molecular analysis of NSP 5a3a over-expression in HN30

**Figure 8:**
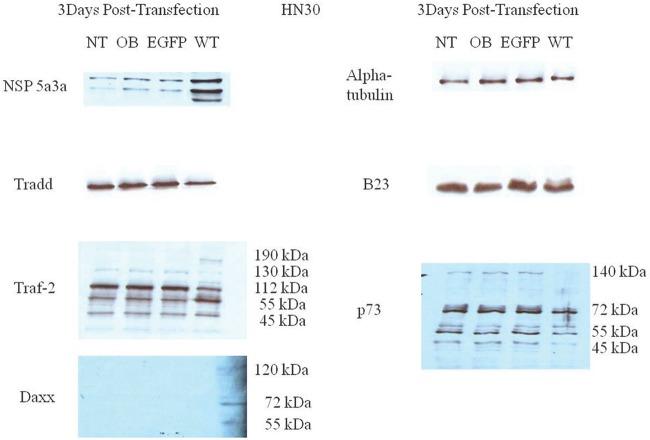
Western Blot analysis of total lysates from asynchronous HN30 cells 3 days post-transfection NT: non-treated, OB: only buffer, EGFP: only pcDNA3.1/CT-GFP vector, NSP 5a3a/EGFP: pcDNA 3.1/CT-GFP and pcDNA 3.0 NSP 5a3a.

Western blot analysis of total lysates of HN30 cells from three days post-transfection along with controls were analyzed to better understand and explain the mechanism that NSP 5a3a may be involved to induce apoptosis as previously described in the HN30 cell line[28]. We considered and examined apoptotic proteins belonging to the caspase family that were not previously investigated in addition to proteins associated with the TNF signaling pathway such as TRAF-2 and TRADD. This was done so in light of the fact that NSP 5a3a over-expression was shown to affect the expression levels of p73 [7] while p73 has been suggested to be regulated by death receptor signaling pathways involving Daxx [72] but also given the fact that potential binding motifs had been previously found on NSP 5a3a for apoptotic proteins associated with TNF/death receptor signaling [7]. TRADD levels seemed apparently the same between treated and controls. TRAF2 levels demonstrated a slight increase in a 190 kDa band in the transfected cells with a slight decrease in the 130 and 112 kDa bands in the treated cells compared to the controls. Though, interestingly there was a moderate increase in the 55 kDa band compared to the controls while a 45 kDa band was comparable between transfected and controls. P73 levels demonstrated a significant decrease in the 140 kDa bands in transfected cells along with a slight decrease in a 55 kDa band and significant decrease in a 45 kDa bands in treated cells compared to the controls. The 72 kDa band remained comparable between treated and controls. B23 levels were seemingly unaffected between treated and controls. DAXX levels were not detected in controls though a band approximately around 72-75 kDa was detected in transfected HN30 cells showing a significant increase compared to controls as well as bands around 55 and 120 kDa were detected in transfected cells but not detected in the controls. Also interestingly, NSP 5a3a levels showed the appearance of a lower band around around 55 to 60 kDa range in the transfected cells (Fig. 8). Cleaved caspase 7, 8, 9, and 1 were not detected in both transfected and controls (data not shown).

### Molecular analysis of NSP 5a3a over-expression in MCF-12a

**Figure 9:**
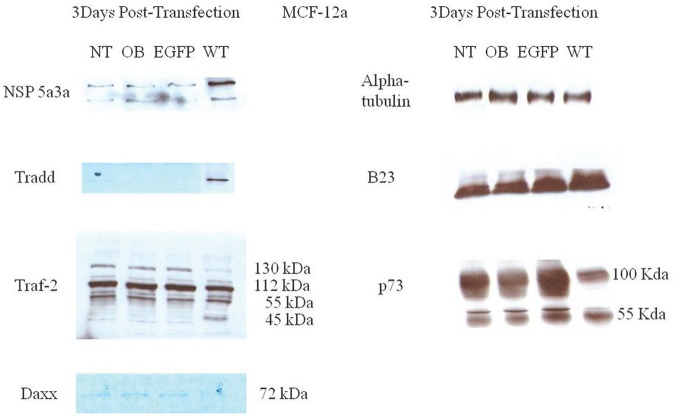
Western Blot analysis of total lysates from asynchronous MCF-12a cells 3 days post-transfection NT: non-treated, OB: only buffer, EGFP: only pcDNA3.1/CT-GFP vector, NSP 5a3a/EGFP: pcDNA 3.1/CT-GFP and pcDNA 3.0 NSP 5a3a.

Western blot analysis of total lysates of MCF-12a cells from three days post-transfection along with controls were analyzed to explain the absence of observed apoptosis in this particular normal cell line in contrast to the observed apoptosis seen in the previous control cell WI-38 when NSP 5a3a was over-expressed [7]. TRADD levels showed a significant increase in transfected cells compared to controls while in controls the TRADD levels were barely if at all detectable. TRAF2 levels showed a slight decrease in the 130 kDa band in transfected cells compared to controls. Bands detected at the 112 kDa and 55 kDa range were comparable between transfected and controls. A slight increase in a 45 kDa band was detected in the transfected compared to controls. P73 levels showed a moderate to significant decrease in a 100 kDa band and a slight to moderate decrease in the 55 kDa in transfected cells compared to the controls. DAXX levels were detected at seemingly low levels around 72-75 kDa in controls though showed a decrease in the transfected cells being undetectable. B23 levels seemed moderately increased in transfected cells compared to the controls. There was no evidence of a lower molecular weight band for NSP 5a3a in the transfected cells (Fig. 9). Again, cleaved caspases 7, 8, 9, and 1 were tested and not detected for both transfected and controls (data not shown).

### Molecular analysis of Co-immunoprecipitations in non-treated and NSP 5a3a over-expressing HN30 cells

**Figure 10:**
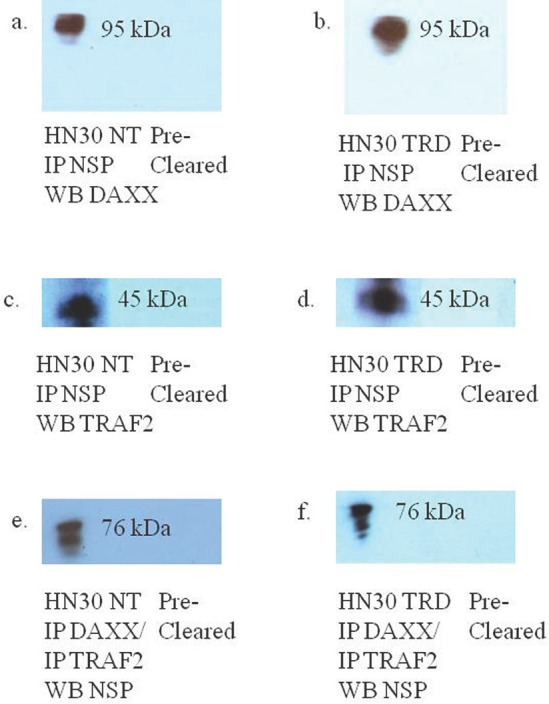
Western blot analysis of co-immunoprecipitations of asynchronous HN30 non-treated and HN30 cells transfected with pcDNA 3.0 NSP 5a3a and pcDNA 3.1/CT-GFP for 3 days NT: non-treated HN30 cells, TRD: HN30 cells over-expressing NSP 5a3a. a. Immunoprecipitation of non-treated HN30 cells using rabbit anti-NSP 5a3a and western blot analysis using rabbit anti-DAXX., b. Immunoprecipitation of HN30 treated cells using rabbit anti-NSP 5a3a and western blot anaylsis using rabbit anti-DAXX., c. Immunoprecipitation of non-treated HN30 cells using rabbit anti-NSP 5a3a and western blot analysis using rabbit anti-TRAF2., d. Immunoprecipitation of HN30 treated cells using rabbit anti-NSP 5a3a and western blot analysis using rabbit anti-TRAF2., e. Immunoprecipitation of non-treated HN30 cells using rabbit anti-TRAF2/DAXX and western blot analysis using rabbit anti-NSP 5a3a, and f. Immunoprecipitation of HN30 treated cells using rabbit anti-TRAF2/DAXX and western blot analysis using rabbit anti-NSP 5a3a.

Western blot analysis of non-treated HN30 cells revealed an interaction between NSP 5a3a and DAXX/TRAF2 as well as an interaction between NSP 5a3a and DAXX/TRAF2 in apoptotic cells over-expressing NSP 5a3a in which we detected a 95 kDa band for DAXX and a 45 kDa band for TRAF2 (Fig. 10). Intensity of the bands for NSP 5a3a, DAXX, and TRAF2 seem slightly more intense in the transfected cells over-expressing NSP 5a3a compared to the non-treated cells though in the case of the NSP 5a3a, which still exhibits the doublet as in the Western blots of the total lysates of HN30 (Fig. 8), the lower molecular weight band seems less intense in the treated cells.

## DISCUSSION

Programmed cell death, also known as apoptosis, is an active daily process within tissues and organs, necessary to ensure the proper functioning of the entire organism being critical for the regulation of development during growth and eventual homeostasis of tissues later in life. This finely tuned balance between cell death and proliferation results from the role of apoptosis in our normal physiological cellular turn-over and maintenance of organ functionality. When this process of apoptosis is unbalanced or unregulated, serious pathological conditions can arise. Whole tissue and organ death have been associated with cardiovascular events such as heart attacks and strokes, while slower cellular death as been associated with particular neurodegenerative diseases such as dementia and Parkinsons [79-80].

Cancer cells seem to have escaped a limited lifespan, evading natural cell turn over and self-destruction by silencing various endogenous executors and stimulators of apoptosis. It seems a fair assessment that the majority of modern day illnesses are associated with dysregulation of the apoptosis, either being over-activated or under-activated depending on the pathology [79-80]. The cancer cells usually suppress apoptosis by three fundamental mechanisms: prevention of pro-caspase activation; neutralization of activated caspases; and silencing of caspase and caspase-like expression at the gene level. Many forms of cancer including leukemia, exhibit over-expression of IAPs (inhibitors of apoptosis) such as c-FLIP and CARD, which can both directly interact with endogenous caspases and regulate their activation in a negative manner, thereby impairing the necessary proteolytic cascade essential for apoptosis. Thus, apoptotic extrinsic pathways involving TNFR/TRAIL-R as well as intrinsic pathways involving cytochrome c and AIF, through caspase dependent and independent mechanisms respectively via the mitochondria can all be abrogated by cancer cells in addition to their ability to alter expression levels and function of anti-apoptotic proteins such as Bcl-2 family members [41, 79-81].

The recent investigative effort in the past decade and half in molecular mechanisms and modulators of apoptosis has generated a greater understanding of this complex biological process and its association with disease in the human body which as a result has pushed for the on-going development of novel strategies targeting various key components of the apoptotic process either in a pro-apoptotic or anti-apoptotic manner. Present on-going apoptotic therapies targeting death receptors have included TRAIL through use of TRAIL-R1/R2 monoclonal antibodies as well as soluble Apo2L/TRAIL, in which most of these therapies have progressed to Phase 1 with exception of the Apo2L/TRAIL. Other apoptotic strategies so far have included targeting caspases such as caspase 1, 3, 6, and 9 through activators of the caspase or inhibitor of the caspase's inhibitor, as well targeting IAPs/SMAC by use of non-peptide small molecules or antisense oligos most of which have remained pre-clinical. There has been also development of small molecule inhibitors as well as use of natural and synthetic compounds in targeting Bcl-2 anti-apoptotic members which have remained mostly preclinical with the exception of a particular antisense oligo in Phase 3 for treatment of leukemia, melanoma, multiple myelomas, and non-small lung cancer [80-81].

There has been development of p53 targeted adenoviral therapies to re-introduce wild type p53 which have reached phase 3 in treatment of Head and Neck cancer including other solid tumors as well as a phase 2/3 combination therapy of advanced squamous cell cancer again using genetically engineered adenovirus ONYX-15 [80]. Though, despite the primary advantages of this system being high level of transduction, high level of gene expression, and being able to infect a broad spectrum of cell types there are setbacks and issues still being addressed and being investigated regarding the issues of developing more cell/tissue specific vectors with minimal host immune/inflammatory responses and cytotoxicity as well as the preventing the generation of a replication competent virus, there still is need to consider and develop further alternative therapeutic approaches via non-viral methods [82].

This subsequent study has revealed an interesting and novel mechanism by which this unique structural protein NSP 5a3a induces apoptosis in HN30 cells lines, by seemingly stimulating extrinsic pathways of apoptosis internally, through two signaling events, one most likely involving p73/DAXX and the other involving TRAF2/TRADD.

HN30 cells over-expressing NSP 5a3a showed clearly cellular stress from apoptosis (Fig. 1) further more evident in the immunostaining of these cells (Fig. 3-6) and as in our previous study, a significant decrease in a higher molecular weight p73 isoform at 140 kDa [7] was detected while there was also significant decrease in a 45 kDa isoform along with a 55 kDa isoform to a lesser extent. Additionally, a 72 kDa isoform was also detected though seemed comparable in levels between controls and treated cells over-expressing NSP 5a3a (Fig. 8). Since the N-terminal truncated p73 isoforms such as delta Np73, ex2p73, and ex2/3p73, have been found to be highly expressed in many cancers and known to possess anti-apoptotic activities [83], it's reasonable that in fact the isoforms of lower molecular weight we see being down-regulated or perhaps degraded are in fact anti-apoptotic which would make sense given the cellular response we observe when over-expressing NSP 5a3a. The fact we used two anti-p73 antibodies (Santa Cruz H-79) and (Santa Cruz S-20) in this study, we would expect a greater degree of detection and perhaps specificity of other isoforms not previously detected by the (Santa Cruz H-79) antibody alone. Typically, the TAp73 isoforms such as: alpha, beta, gamma, and delta are linked to cell cycle arrest and apoptosis though which cell fate is determined depends on the cell type and also activating p53 target genes are activated and in which cell type [84]. Usually, apoptosis is evident by an up-regulation of a TAp73 isoform as seen with TAp73 alpha and Tap73 beta [84]. Previously, it was suggested that the 140 kDa along with the 72 kDa isoforms may be a result of some post-translational modification of a p73 isoform, since higher molecular weight isoforms of p73 have been identified around 75, 90 and 140 kDa in specific regions of adult human brain [85]. Since it has been shown that p73 can be ubiquitinated, in particular TAp73a and deltaNp73 isoforms can be ubiquitinated by Itch, which results in proteasomal-dependent degradation of these p73 isoforms [86-87], it is plausible that the higher molecular weight of 140 kDa we observe may be the apoptotic form of p73, possibly being p73a that is polyubiquitinated in the controls while in the cells over-expressing NSP 5a3a, there is a decrease in its polyubiquitinated form resulting in more of the apoptotic isoform being present and stable to promote its activity. While MCF-12a cells over-expressing NSP 5a3a did not exhibit cellular stress being apoptotic in appearance as evident in (Fig. 2) and the immunostaining (Fig. 3-6), the MCF-12a cells showed a 55 kDa isoform being down-regulated in cells over-expressing NSP 5a3a (Fig. 9) though we cannot be certain if in fact this isoform is the same as the one observed in the HN30 cells, but is plausible since both apoptotic and anti-apoptotic functions have been assigned to these deltaNp73 isoforms [83,88] and interestingly, we observed a 100 kDa isoform being down-regulated in the MCF-12a cells over-expressing NSP 5a3a (Fig. 9), which again may be a post-translational modification such as ubiquitination of an anti-apoptotic form of p73 though in all cases, until more specific antibodies for such post-translational modifications are tested, we cannot be sure of the exact nature of the type of covalent modification to these isoforms. The same plausibility would hold also in the case of sumoylation of p73, since it has been reported that for example SUMO-1 does covalently modify both full length TA p73a and deltaNp73a isoforms. While usually sumoylation has been associated with different cellular processes such as transcriptional regulation, subcellular transport regulation, influence protein stability and protein function in both cell cycle and apoptosis, in the case of p73a, its sumoylation has been associated with alteration in its subcellular localization and subsequent degradation [89-90].

In our previous study, we had found by use of a motif finder at http://motif.genome.jp/ a potential interaction site in NSP 5a3a with DAXX at at amino acid site: 276-330 and thus wanted to confirm if such an interaction existed between the two proteins also given that fact that DAXX was known to be involved in the death doman receptor signaling pathway involving Fas [91]. Co-immunoprecipitations of HN30 non-treated and treated cells over-expressing NSP 5a3a revealed an interaction between NSP 5a3a and DAXX while seemingly more intense in treated cells with a band at approximately 95 kDa (Fig. 10). DAXX has been reported to act as a transcriptional repressor of many DNA-binding proteins [92-93], while it has been linked to both apoptotic and anti-apoptotic roles [91, 94] where silencing of DAXX sensitized cells to both Fas and stress-induced apoptosis [95]. In regards to its apoptotic role, DAXX has been associated with FAS signaling, linking that signaling to the JNK pathway through ASK1 activation. Normally, DAXX is predominantly nuclear acting as a transcriptional repressor in the nucleus though under apoptotic conditions in which it mediates its effect through ASK1, it shuttles to the cytoplasm thus promoting caspase-independent apoptosis is promoted [96-99]. Interestingly, DAXX has been reported to interact with P73 acting as a transcriptional co-repressor of p53 and its members, by which this regulation is modulated by PML (promyelocytic leukemia protein) occurring in nuclear PODs (PML oncogenic domains) [100]. HN30 cells over-expressing NSP 5a3a showed a significant increase in DAXX with observation of a band between 72-75 kDa and a band between 120-130 kDa while being undetectable in controls (Fig. 8), and in fact similar distinct migratory DAXX forms have been reported in which a 70kDa form was known to be non-phosphorylated and known to have transcriptional co-repressor activity as also a 97 kDa and 120 kDa bands have been identified relating to DAXX while the 120 kDa was revealed to be a result in part of a post-translational modification involving phosphorylation [101]. It is likely the 95 kDa may also be in part to a post-translational modification in the conditions we observed. A similar band of 72-75 kDa was observed in controls of the MCF-12a though fairly weak and being nearly undetectable in MCF-12a cells over-expressing NSP 5a3a (Fig. 9). While we also detected a much lower band around 55 kDa in the HN30 treated cells we cannot be certain if this represents a non-specific band or perhaps some fragment of DAXX. Immunostaining of HN30 cells over-expressing NSP 5a3a showed a seemingly increased distribution of p73 from the nucleus into the cytoplasm, though not as quite as diffuse into the cytoplasm as the MCF-12a cells over-expressing NSP 5a3a (Fig. 4). Since the antibody used for the immunostaining for p73 was the rabbit polyclonal p73 (Santa Cruz H-79) and even though according the manufacturer the antibody should be able to recognize not only the apoptotic TAp73 isoforms (α,β,γ,δ,ε,η,ζ) [83] if expressed but also the anti-apoptotic forms ex2p73 (α,β,γ,δ,ε,η,ζ) and ex2/3p73 (α,β,γ,δ,ε,η,ζ) [83], we were unable in our previous study to detect any bands that corresponded to those isoforms. Even though it is possible that this particular antibody may behave differently given the application it is used for thus allowing for different specificity and sensitivity, we cannot be sure if what we see by the immunostaining is solely the higher molecular weight isoform of 140 kDa in the case of the HN30 cells over-expressing NSP 5a3a. It's been reported that TAp73 can localize in both the cytoplasm and nucleus while the deltaNp73 forms tend to localize mostly in the nucleus as seen in-vivo in breast and non-small lung cancer [102-103]. TAp73 and deltaNp73 have been seen mostly in nuclear compartments in-vitro though there has been variation in its localization with limited cytoplasmic localization being mostly nuclear to even being exclusively cytoplasmic as seen in medullobastoma cell lines [104]. It is possible that we are observing an increased translocation of p73 from the nucleus to the cytoplasm due to an increase ubiquitination of the a particular p73 isoform/s, since it's been shown that p73 is ubiquitinated in the nucleus, this process regulated by PML and thus then shuttles to the cytoplasm for proteasomal degradation though cannot exclude should this particular p73 isoform/s may be instead sumoylated which can lead to proteasomal degradation as well [86, 105] as also transcriptional repression involving DAXX in PODs [100]. Then again, p73 has been known to interact with DAXX and it has been suggested that p53/p73 may potentiate DAXX's pro-apoptotic function when p53/p73 associate with DAXX in the PODs of the nucleus [106]. Since we do not know the behavior of DAXX during over-expression of NSP 5a3a nor its interaction with p73 if still occurring in PODs regions of the nucleus or in other compartments, further study in needed to elucidate this likely signaling interaction involving p73 and DAXX. Thus, it is possible that NSP 5a3a may be assisting DAXX to regulate particular p73 isoforms perhaps in a negative manner in the PODs while enhancing its apoptotic activity with other p73 isoforms depending on the cell type, cellular events in action and compartments in which they find themselves, yet also assisting DAXX to interact with other apoptotic proteins most likely in the ASK1-JNK pathway in parallel. Given, that in our previous study we had detected a Parp cleavage product of 89 kDa in HN30 cells over-expressing NSP 5a3a, which typically is associated with caspase activation in-vitro usually by caspase 3 as well as other caspases if caspase 3 is not available [107], we could not confirm any such activation and having been already documented of the existence of Parp cleavage associated with caspase 3 independent activation [108-109] we further tested in this study if there was activation of other canonical caspases such as caspase 9, 8, 7 and 1, in which we were not able to detect any cleaved products hence caspase activation which seems to point more in favor of a caspase independent mechanism, which has been associated with DAXX activation of the ASK1-JNK pathway [110]. Though Parp cleavage is known as classical apoptotic hallmark [111] and it has been documented that DAXX ectopic over-expression has been associated with caspase dependent activation by FAS and TRAIL induced apoptosis [112] one cannot exclude that depending on the cell system and apoptotic conditions being investigated that perhaps through NSP 5a3a over-expression, DAXX may be involved in a caspase independent activation of JNK pathway with Parp cleavage since Parp cleavage has been seen independent of caspase activation [113-115]. Furthermore, given that an interaction between NSP 5a3a and a higher molecular weight of DAXX at 95 kDa was confirmed while modulation in a 72-75 kDa band as well as a 120-130 kDa was detected in the HN30 treated cells, this may indicate that NSP 5a3a preferentially interacts with a particular post-translational form of DAXX which may contribute to regulation and stability of the other DAXX un-modified and modified forms post-translationally.

When we examined for other members involved in the ASK1-JNK pathway, we found by use of ELM motif finder at http://elm.eu.org/ potential sites on NSP 5a3a for interaction with TRAF2 at amino acid sites: 376-379 (TTQE) and 455-458 (TLEE) as well as able to identify another nine potential TRAF2 consensus interaction sequences being the (SXXE) motif [116] in NSP 5a3. We thus were able to confirm an interaction between NSP 5a3a and TRAF2 in both HN30 cells over-expressing NSP 5a3a and non-treated cells, identifying in both cases a band approximately 45 kDa (Fig. 10). HN30 cells over-expressing NSP 5a3a showed a slight decrease in 112 and 130 kDa isoforms compared to controls with a slight increase in a 190 kDa isoform and more moderate increase in a 55 kDa isoform compared to controls (Fig. 8). We also observed a 45 kDa isoform which showed no apparent change in levels between treated and controls (Fig. 8). Similar bands were identified in MCF-12a cells though with a different pattern of expression in which a 130 kDa isoform was slightly decreased in MCF-12a cells over-expressing NSP 5a3a while the 112 kDa and 55 kDa isoforms remained apparently the same between treated cells and controls (Fig. 9). The lower molecular weight 45kDa isoform seemed slightly increased in MCF-12a cells over-expressing NSP 5a3a (Fig. 9). We can expect to see a broad range of isoforms since we used a rabbit polyclonal TRAF2 (Novus NB100-56173) antibody that has been documented to be able to detect a broad range of TRAF2 isoforms again depending on cell type and conditions. TRAF2 has been known well to be involved in the NF-KB pathway, both as positive regulator of the canonical pathway and as a negative regulator as well, in which depending on cell type and signaling intermediates and adaptors available it can favor or not cellular proliferation and survival [54, 117]. Though, it has been reported that TRAF2 can be involved in JNK activation leading to apoptosis by TNFa induction [118], by which TRAF2 can be polyubiquitinated at K63 resulting in its degradation and translocation to the cytoplasmic insoluble membrane fraction and cytoskeletal one as well [54, 119-120]. Interestingly, we see modulation in protein levels of TRAF2 of both high and low molecular weight isoforms in the HN30 cells over-expressing NSP 5a3a, in which the higher molecular weight isoforms may be in fact post-translational modifications of TRAF2 lower weight isoforms resulting in polyubiquitination and given the fact we see an increased immunostaining for TRAF2 in the cytoplasm and association with likely apoptotic bodies as well (Fig. 5), it would seem plausible to support the idea that TRAF2 may be involved in mediating apoptotic signaling thru interaction of NSP 5a3a which could serve as a signaling scaffold for TRAF2 isoforms. It's been suggested that TRAF2 localization in the nucleus may be linked to transcriptional regulation while in the cytoplasm it may be linked to signal transduction [121]. In contrast, MCF-12a cells exhibited a more nuclear staining in the cells over-expressing NSP 5a3a compared to its controls though with lesser staining in the cytoplasm compared to the treated HN30 cells (Fig. 5). Even though both cell types expressed similar isoforms, it seems that in the MCF-12a the over-expression of NSP 5a3a did not induce the same intracellular stress response resulting in apoptosis, though in the case of the MCF-12a, the TRAF2 may be involved in activation or regulation of proteins involved in the NF-KB pathway for example, leading to either

prosurvival or cell cycle arrest. This we cannot be sure of in the case of the MCF-12a, since we would need further study of the cell cycle during over-expression of NSP 5a3a and analysis of NF-KB pathway members as well. Even though, NF-KB signaling has been found to be involved in apoptosis depending on the nature of the apoptotic stimuli [122-123] it usually involves the repression or down-regulation of target genes including TRAF2 [124], which ultimately makes more favorable the implication of TRAF2 in association with NSP 5a3a in the induction of apoptosis in HN30, whether the signal is being mediated through ASK1 leading to JNK activation or even through RIP which can lead to JNK activation and apoptosis also [125-126].

Another TNFR signaling protein known to interact with TRAF2 is TRADD particular to the TNFR1 pathway which can depending with which signaling and adaptor proteins it interacts with, it can either be involved in NF-kB activation thru binding with TRAF2 or activation of apoptosis thru interaction with FADD [127-128]. While the levels of TRADD seemed comparable between controls and HN30 cells over-expressing NSP 5a3a (Fig. 8) there was an evident change in the distribution of TRADD as indicated by the immunostaining (Fig. 6) being more nuclear as well as more cytoplasmic in treated cells along with association with apoptotic bodies. In contrast, a significant increase in TRADD expression was detected in MCF-12a cells over-expressing NSP 5a3a while being undetectable in the control (Fig. 9) though we still observed its expression in controls by immunostaining indicating a likely difference in sensitivity both in the behavior of the antibody used as well as the application. There seemed to me a more diffuse staining of TRADD into the cytoplasm of the MCF-12a treated cells compared to the controls, while still be mostly nuclear in both cases. While TRADD depending on its location in the cell can induce apoptosis by different mechanisms, either through a FADD/caspase 8 pathway in the cytoplasm or through caspase 9 activation when in the nucleus [129], in both cases we exclude such pathways since we were unable to detect caspase 8/9 activation in the HN30 treated cells. The fact we see appreciable expression levels of both TRADD and TRAF2 in the controls of the HN30 cells is of interest since it has been reported that the TNFR1-TRADD-TRAF2-RIP pathway is switched on in head and neck cell carcinoma leading to NF-kB constitutive activation and hence cell survival and proliferation [130]. Given this cellular behavior of the head and neck carcinoma in which this particular signaling pathway is already activated, it is plausible that NSP 5a3a may be altering this signaling pathway in favor of apoptosis through use of TRAF2 and TRADD. While in the MCF-12a cells, not showing the same cellular stress as in the HN30, NSP 5a3a-TRAF2 could be interacting with TRADD in promoting cell growth arrest or survival through the NF-kB pathway. Since we do not know the dynamics of interaction between TRADD and TRAF2 in the apoptotic conditions we observed, we cannot be sure if there is a novel interaction between TRADD and TRAF2 mediated by NSP 5a3a that is involved in the promoting the apoptosis thru the ASK1/JNK pathway. Even though it has been shown that TRADD-TRAF2 interaction in the TNFR1 signaling pathway leads to NF-kB activation and cell survival, such that TRAF2 is not essential for TNFR1 apoptotic signaling [117, 131] we cannot exclude a possible undefined interaction between NSP 5a3a/TRAF2 with TRADD and other signaling and adaptor proteins that would allow the cells to die by apoptosis. Interestingly, though there no evident change in expression in the B23 levels in H30 cells over-expressing NSP 5a3a, as seen reported in our previous study [7], we did observe a moderate increase in B23 levels in the MCF-12a cells over-expressing NSP 5a3a which may be contributing to the observed non-stressed and non-apoptotic state of these cells since B23 over-expression has been associated with cell cycle arrest and proliferation [132-133] though again we cannot be sure if the cells are in fact arrested in particular phase of the cell cycle though given the expression of B23 along with TRADD and TRAF2 in the MCF-12a. Though, one cannot rule out that in this particular normal cell line, there may be a short circuit or interruption of induction of apoptosis and instead favoring either cell growth arrest or proliferation.

The HN30 cells over-expressing NSP 5a3a showed by immunostaining an increase in cytoplasmic localization compared to controls while also being associated with likely apoptotic bodies. In contrast, the MCF-12a cells both treated and controls showed still mostly nuclear staining without any significant change in localization of NSP 5a3a when over-expressed (Fig. 3). Similarly, there was also an association of TRADD and TRAF2 with or nearby apoptotic bodies in HN30 cells over-expressing NSP 5a3a (Fig. 5-6). While typically, apoptotic bodies and apoptotic blebs tend to be more associated with late stage apoptosis [134-135] being able to contain nuclear proteins, DNA, and RNA molecules [136] it would make sense that such proteins like: NSP 5a3a, TRADD and TRAF2, can exist and be included in such apoptotic bodies in such experimental conditions, which raises the question if these apoptotic bodies may be involved in extracellular induction of apoptosis with neighboring cells since it has been reported that apoptotic bodies can modulate intracellular communication [136-137]. Even though, mostly histone proteins 1-4 have been found inside apoptotic bodies as in the case of lymphoblasts [138], perhaps depending on the type of apoptotic induction and cell type, TNFR signaling proteins could remain encapsulated in these apoptotic bodies as in our case, and making it more intriguing is the fact that death receptor ligands, such as FasL and TRAIL have been found expressed on tumor microvesicles, albeit smaller than apoptotic bodies in size [139], in head and neck carcinoma from patients [140]. It opens possibilities if in the apoptotic scenario we present, that NSP 5a3a, TRAF2 and TRADD may be mediating apoptotic signals to other cells thru surface death receptor TNFR via apoptotic bodies or blebs that are formed. While, bleb formation has been also been found in early stages of apoptosis, being termed “marginal blebbing”, which leads to more advanced stages of actual formation and separation of apoptotic bodies from dying cells, caspase activity was found associated with blebbing and apoptotic body formation, related to activation of at least either caspase 3 or 6 with also an increase in cytochrome c release from the mitochondria, this seen by TNFa induced apoptosis in Hela cells [141]. Though, again, we were not able to detect any caspase activity given the caspases we tested nor did we see any changes in cytochrome c levels as demonstrated in our previous study in HN30 treated cell [7].

We demonstrated in this subsequent study that NSP 5a3a seems to be triggering apoptosis and mediating its effects through 2 distinct axis points in the TNFR-1 signaling pathway. One axis point involves p73 isoforms and DAXX while the other axis point involves TRAF2 with possibly TRADD. How NSP 5a3a may modulate any possible cross-talk between these signaling axis points, how these apoptotic adaptors interact with each other and with other signaling proteins, both in apoptotic and anti-apoptotic pathways depending on the cell type still warrants further investigation. It is known that scaffold proteins enable a high degree of specificity between interacting proteins and substrates as well being catalysts in signaling pathways such as the MAPK/JNK pathways [142-143], thereby being able to function as molecular assembly platforms, allowing various domains of proteins to interact in a flexible manner [143]. Besides being involved in regulating cellular communication at cell-cell signaling junctions, coordinating assembly sequence interactions, scaffold proteins also have been found to participate in intracellular signaling in regulation of kinase cascade pathways, as well being themselves substrates for regulation in which a particular pathway may be switched on or off, and lastly being involved in regulatory feedback loops in a signaling pathway [143]. Interestingly, when we examined NSP 5a3a and compared its domain structure to recently identified scaffold proteins involved in MAPK/JNK signaling, such as those proteins belonging to the JIP family, JIP1, JIP2, JIP3, and SPAG9 [144-145], we found an intriguing feature that NSP 5a3a shared with the JIP family proteins. Even though NSP 5a3a lacked particular domain features of the JIP family such as the JNK-binding domain, we did find potential MAPK/JNK docking sites known as the D-site in the NSP 5a3a amino acid sequence between amino acids: 361-515. The D-site is characterized by the following consensus sequence: (R/K)_3-5_ (X)_1-5_ (Φ-X-Φ) where there is a group of basic residues followed by a spacer region and then a hydrophobic submotif [146]. In light of the expanding multiple roles and functions of structural scaffolding proteins in normal and pathologic physiological states, it would be of great interest in future studies to better understand how NSP 5a3a may regulate and be regulated itself involving these recently discovered axis points along the ASK1/JNK pathway and its possible molecular coordination of other signaling pathways that may cross-talk and converge with the ASK1/JNK pathway. Is it also of interest to investigate better how these axis points are coordinated between each other and how they may cross-talk, since it was recently reported that there is a cross-talk between p73 isoforms and c-JUN of the JNK pathway which can either lead to apoptosis or proliferation [147].

## MATERIALS AND METHODS

### Cell Lines and Tissue Culture

We used following cell lines in this study: HN30 (Head and Neck carcinoma) and MCF-12a (Normal Breast Epithelial). All these cell lines were obtained from ATCC and cultured at conditions recommended by ATCC except for the HN30 cell line which was a kind gift from Dr. George Yoo of the Department of Otolaryngology, Head and Neck Surgery, Wayne State University and Karmanos Cancer Institute, Detroit, Michigan.

### DNA Transfection and Western Blot Analysis

Asynchronous HN30 and MCF-12a cells were seeded in 6 well-plates and transfected and optimized with Fugene HD (Roche) according to the manufacturer's protocol using 2 ug of plasmid DNA per well. Plasmids used for transfection were the following: pcDNA3.1/CT-GFP (invitrogen) and pcDNA 3.0 NSP 5a3a. The NSP 5a3a cDNA had been cloned from a previous work [1]. The pcDNA3.1/CT-GFP vector was used to monitor transfection efficiency for all cell lines in the study.

HN30 and MCF-12a cells at three days post-transfection were harvested mechanically with a scraper, spun and washed twice with PBS 1x after which cells were prepped for western blot analysis. Cell pellets were lysed using a total lysis buffer (50mM Tris-Cl pH 7.4, 5mM EDTA, 250 mM NaCl, 50 mM NaF, .1% Triton X-100, .1 mM Na3VO4, final volume with dH2O) for 15 minutes on ice then for 30 minutes on a rotator at 4 degrees. Lysates were spun down at 13,000 rpm for 15 minutes at 4 degrees after which supernatants were collected and protein concentration was determined by Bradford Assay. A total of 30 ug of total protein was loaded for each sample and separated on 7% SDS-PAGE or 15% gel depending on the molecular weight of the proteins to be separated then followed by transfer onto Whatman Protran Nitrocellulose Transfer Membrane for 1.5 hours at 70 volts at 4 degrees.

Membranes were pre-blocked overnight at 4 degrees in 5% milk buffer TTBS. Next day, membranes were cut and incubated with primary rabbit polyclonal NSP5a3a (Novus NB100-517A) at 1/500, primary rabbit polyclonal p73 (Santa Cruz H-79) at 1/200, primary goat polyclonal p73 (Santa Cruz S-20) at 1/200, primary mouse monoclonal alpha-tubulin (Invitrogen) at 1/5000, primary rabbit polyclonal TRADD (Cell Signaling) 1/1000, primary rabbit polyclonal TRAF2 (Novus NB100-56173) at 1/1000, primary rabbit polyclonal DAXX (Cell Signaling 25C12) at 1/1000, primary rabbit polyclonal cleaved caspase 7 (Cell Signaling D198) at 1/x, primary rabbit polyclonal cleaved caspase 8 (Cell Signaling 18C8) at 1/x, rabbit primary polyclonal cleaved caspase 9 (Cell Signaling D3C5) at 1/x, primary rabbit polyclonal caspase 1 (Cell Signaling) at 1/x or primary mouse monoclonal B23 (Sigma) at 1/500 in 5% milk buffer TTBS for 2 hours at RT.

Membranes were then washed 4 times at 15 minute intervals in .1% Tween in PBS 1x then were incubated with secondary anti-rabbit HRP (Amersham Biosciences) at 1/5000, secondary anti-goat HRP (Amersham Biosciences) at 1/5000 or secondary anti-mouse HRP (Amersham Biosciences) at 1/5000 in 5% milk buffer for 1 hour at RT. Membranes were then again washed 4 times at 15 minute intervals each and then exposed for 3 minutes to ECL Chemiluminescent Detection Reagent (Perkin Elmer) to be developed on Kodak x-ray film.

### Co-immunoprecipitations and Western Blot Analysis

Asynchronous HN30 cells were plated in 60 mm Dishes so they would be approximately ~60% confluent next day for transfection. The day of transfection, HN30 cells were transfected with recommended amount of pcDNA 3.0 NSP 5a3a and pcDNA 3.1/CT-GFP using Fugene HD (Roche) as indicated by the manufacturer. Three days after transfection, HN30 cells treated with pcDNA 3.0 NSP 5a3a and pcDNA 3.1/CT-GFP were harvested along with non-treated HN30 cells using a mechanical scraper. The cells were washed twice with PBS 1x after which Total lysis buffer was used to lyse cells. While cells were lysed, 100 ul of Protein A/G sepharose beads (Pierce) were washed with 900 ul cold lysis buffer, spun down for ~30 seconds at 11,000 rpm, then washed again with 1 ml of cold lysis buffer, spun down and then were resuspended in 100 ul of cold lysis buffer and kept at 4 degrees until needed. Approximately, 500-800 total protein for both treated and non-treated lysates were incubated with 25 ul of pre-washed protein A/G beads for 1 hour rotating at 4 degrees to pre-clear the lysates. After incubation with pre-washed beads, the lysates were spun down for 30 seconds at 11,000 rpm at 4 degrees, and then supernatants were transferred to prepped cold eppendorf tubes while the pre-cleared beads were saved at 4 degrees. The supernatants for both treated and non-treated were then split into equal amounts of total protein and were incubated with either 5 ug of rabbit polyclonal NSP 5a3a (Novus), rabbit polyclonal DAXX (Cell Signaling), or rabbit polyclonal TRAF2 (Novus) for 1 hour rotating at 4 degrees. After supernatants incubated with their respective antibodies, approximately 25 ul of pre-washed protein A/G beads were added and allowed to incubate with the lysates plus antibodies rotating at 4 degrees overnight. The next day, the supernatants were spun down for 30 seconds at 11,000 rpm after which the supernatants were removed and the beads were washed 5 times with 500 ul of cold total lysis buffer. Then, 25 ul of Laemli 1x buffer with no beta-mercaptoethanol were added to the beads and not boiled after which the co-ips were loaded on a 7% SDS-PAGE gel and the membranes were immunoblotted accordingly with rabbit polyclonal NSP 5a3a (Novus) at 1/500, rabbit polyclonal DAXX (Cell Signaling) at 1/1000, or rabbit polyclonal TRAF2 (Novus) at 1/1000. Along with pre-cleared and pre-washed protein A/G beads, also primary rabbit IgGs was loaded on the gel as control.

### Immunofluorescence Staining

Three days before immuno-staining, HN30 and MCF-12a cells were plated at a confluency of 150,000 cells per well of a 6 well plate and grown onto sterile coverslips (22x22x1mm). The next day cells were either transfected with pcDNA 3.0 NSP 5a3a using Fugene HD (Roche) or left un-treated. Three days post-transfection, treated and non-treated cells were washed once with serum free medium for 2-3 minutes at room temperature and fixed in 4% paraformaldhyde for 20 minutes and permeabilized for 10 minutes at room temperature. Cells were pre-blocked for 1 hour at room temperature with 8% BSA in PBS 1x, then cells were incubated with either with rabbit polyclonal NSP 5a3a (Novus NB100-517A) at 1/500, rabbit polyclonal TRADD (Cell Signaling) at 1/1000, rabbit polyclonal TRAF2 (Novus NB100-56173) at 1/1000, or rabbit polyclonal p73 (Santa Cruz H-79) at 1/200 dilution in 1% BSA overnight at 4°C. Next day, the primary antibody was removed and then after 3-5 washes in PBS 1 x for 3-5 minutes each wash, cells were incubated with anti-rabbit FITC (Invitrogen) at 1/700 dilution in 1% BSA in PBS 1x and incubated for 1 hour at room temperature. Cover slips were mounted on glass slides with vector shield plus DAPI and edges of cover slips sealed with nail hardener. Images were acquired on an Olympus IX81 Confocal microscope with a 60x oil objective. Single plane images were taken with a sensicam qe and acquired using the imaging software slidebook 4.1.0. Images were taken in one plane at a .5 um thickness.

### Light Microscopy

Asynchronous HN30 and MCF-12a cells, three days post-transfection were observed for morphological changes using Zeiss Axiovert 25 microscope with 10x PH1 Zeiis objective.
